# Peculiar Form of Epilepsy

**Published:** 1850-01

**Authors:** F. H. Hamilton

**Affiliations:** One of the attending surgeons to the “Buffalo Hospital of the Sisters of Charity”


ART. II.—*Peculiar form of Epilepsy.* Reported by Dr. F. H. HAMILTON, one of the attending surgeons to the "Buffalo Hospital of the Sisters of Charity."

C. B——, printer, of Rochester, N. Y., entered the Hospital as my private patient, Jan. 16th, 1849. The following is an abridgment of a letter which he addressed to me, detailing the history and progress of his malady:—

"I am now twenty-three years old. At the age of three years I fell from the bed, and struck my head upon the spot where phrenologists locate the organ of 'hope.' The physician who examined it said it was a mere bruise. The wound, however, did not close in two years, but a sinus was formed under the scalp, extending from the seat of the original injury, to a point two inches nearer the ear. Finally it opened at this latter point, and then the first wound healed. In one year more, it healed also at the lower opening. Now I became affected with a kind of spasm and vertigo. The spasms were always preceded by a sensation similar to that produced by a spider running from the ear to the original wound. By a course of emetics and purgatives, I obtained some relief, at the age of seven years. Eight years since, I discovered a depression at the point of injury, which I think, by my frequent pressing upon it, has much increased in breadth and depth.

But to speak more particularly of the manner in which it affects me. From the age of five to nine years, on the occurrence of the spasm, I was thrown instantly upon my back, with my feet and hands lifted perpendicularly into the air; and I laughed constantly until the spasm ceased. Since then, unusual mental exertion renders me almost helpless, from extreme weakness, and my brain is confused, but the spasms are not so severe, or of the same character. Now if I press upon either of the old scars, I can induce this condition, and a nervous sensation extends from the point pressed upon, down my neck, shoulders, &c. If the pressure is continued, it produces, in fifteen minutes, copious salivary, urinary and alvine evacuations. If spasms occur, my vision is affected, and objects appear much more distant than they actually are. If I am walking, under its influence, my speed is immediately involuntarily accelerated, and perhaps, in a moment afterwards, my progression is in like proportion retarded. If the spasms are chiefly in the right side, I walk obliquely to the right, if in the left side, I walk obliquely in the opposite direction. In this condition, I cannot give correct utterance to my thoughts, but I think one thing and speak another. Sometimes when engaged in type setting, I commit gross blunders, and then not from accident or dullness of intellect, but because I am impelled or determined to do it. Recently, after having supped, and while yet sitting at the table, and knowing that such was the fact, I said, 'let us ask a blessing,' and I proceeded to do so, but was arrested in the middle of the service, by the impulse having suddenly ceased. Again, I was splitting wood in the rear of the house, when I was taken by a spasm, and forthwith I started, pell-mell, for the street, a distance of six rods, with no object in view, yet with the axe raised as if in the act of striking.

When I reached the street, the excitement ceased, and I returned quietly, but greatly exhausted."

January, 1849, I operated upon Mr. B., at the Hospital, he having placed himself under my care for that purpose.

The operation consisted in nearly circumscribing each of the cicatrices, separately, by a circular incision extending to the bone, and then dissecting it up clean from the cranium, leaving the circular flap thus elevated, attached only at one point, of about an inch in breadth, through which it might continue to derive its support. My object was twofold: first, to cut off, as completely as possible, the nervous communication between these cicatrices, and the general system, and second, to afford me an opportunity to trephine, if the skull should be found to be depressed. There was, however, no evidence that the skull had ever been injured; I therefore completed the operation by simply replacing the flaps. He was immediately, and for a short time, relieved of nearly all the unpleasant symptoms, from which he had so long suffered. In about two weeks he returned home. The following is a summary of the letter which he has since addressed to me:—

"*Dear Sir:*—I have delayed writing to you thus long, that I might speak more definitely of my case, and of the benefits received from the operation which you made. I am happy in now being able to say that I am greatly benefitted; indeed, I do not hesitate to say that I am permanently cured. It is now three months since the operation, and I feel like a new man. During the healing process, I was almost in despair as to any favorable results; many of my old symptoms returned. But, when, in about five weeks, the wounds had entirely healed, the unwelcome symptoms again disappeared, and they have not returned. The upper cicatrix is soft and pliable like pulp.

My mind is not now, as formerly, confused and distracted; I have, in consequence, been able to make a desirable editorial connection, and my future prospects are brightened. For these priceless benefits, please accept my thanks. Give, also, my thanks to sister Ursula, for her, and the Sisters' kind attentions to me during my brief stay with them.

Your sincere friend,

March 26th, 1849. C. B."

